# Supplementary material for: MiR-155-5p Attenuates Vascular Smooth Muscle Cell Oxidative Stress and Migration via Inhibiting BACH1 Expression
Source: Biomedicines. 2023 Jun 9;11(6):1679. doi: 10.3390/biomedicines11061679 (PMC10295705; doi:10.3390/biomedicines11061679)
Supplement: Supplementary file 1 [file biomedicines-11-01679-s001.zip › biomedicines-2313947-supplementary.pdf]

Table S1. Primers for RT-PCR analysis in rats.

Name	Primer	Sequence
BACH1	Forward	GCGGACTTTCACAACTCTCA
	Reverse	CTGAGGCTCTGCGACGTAAT
GAPDH	Forward	GGAAAGCTGTGGCGTGAT
	Reverse	AAGGTGGAAGAATGGGAGTT
miR-155-5p		CGTTAATGCTAATTGTGATAGGGGT
U6		CTCGCTTCGGCAGCACA
